# Heat-Responsive Proteomics of a Heat-Sensitive Spinach Variety

**DOI:** 10.3390/ijms20163872

**Published:** 2019-08-08

**Authors:** Shanshan Li, Juanjuan Yu, Ying Li, Heng Zhang, Xuesong Bao, Jiayi Bian, Chenxi Xu, Xiaoli Wang, Xiaofeng Cai, Quanhua Wang, Pengcheng Wang, Siyi Guo, Yuchen Miao, Sixue Chen, Zhi Qin, Shaojun Dai

**Affiliations:** 1 Development Center of Plant Germplasm Resources, College of Life Sciences, Shanghai Normal University, Shanghai 200234, China; 2 Alkali Soil Natural Environmental Science Center, Northeast Forestry University, Key Laboratory of Saline-alkali Vegetation Ecology Restoration, Ministry of Education, Harbin 150040, China; 3 College of Life Sciences and Agriculture and Forestry, Qiqihar University, Qiqihar 161006, China; 4 College of Life Sciences, Henan Normal University, Xinxiang 453007, China; 5 Shanghai Center for Plant Stress Biology, Chinese Academy of Sciences, Shanghai 201602, China; 6 Institute of Plant Stress Biology, State Key Laboratory of Cotton Biology, Department of Biology, Henan University, Kaifeng 475004, China; 7 Department of Biology, Genetics Institute, Plant Molecular and Cellular Biology Program, Interdisciplinary Center for Biotechnology Research, University of Florida, Gainesville, FL 32610, USA

**Keywords:** heat response, heat-sensitive spinach variety, proteomics, ROS scavenging

## Abstract

High temperatures seriously limit plant growth and productivity. Investigating heat-responsive molecular mechanisms is important for breeding heat-tolerant crops. In this study, heat-responsive mechanisms in leaves from a heat-sensitive spinach (*Spinacia oleracea* L.) variety Sp73 were investigated using two-dimensional gel electrophoresis (2DE)-based and isobaric tags for relative and absolute quantification (iTRAQ)-based proteomics approaches. In total, 257 heat-responsive proteins were identified in the spinach leaves. The abundance patterns of these proteins indicated that the photosynthesis process was inhibited, reactive oxygen species (ROS) scavenging pathways were initiated, and protein synthesis and turnover, carbohydrate and amino acid metabolism were promoted in the spinach Sp73 in response to high temperature. By comparing this with our previous results in the heat-tolerant spinach variety Sp75, we found that heat inhibited photosynthesis, as well as heat-enhanced ROS scavenging, stress defense pathways, carbohydrate and energy metabolism, and protein folding and turnover constituting a conservative strategy for spinach in response to heat stress. However, the heat-decreased biosynthesis of chlorophyll and carotenoid as well as soluble sugar content in the variety Sp73 was quite different from that in the variety Sp75, leading to a lower capability for photosynthetic adaptation and osmotic homeostasis in Sp73 under heat stress. Moreover, the heat-reduced activities of SOD and other heat-activated antioxidant enzymes in the heat-sensitive variety Sp73 were also different from the heat-tolerant variety Sp75, implying that the ROS scavenging strategy is critical for heat tolerance.

## 1. Introduction

Global warming has adverse effects on crop yield [1,2]. Heat stress limits plant growth, development and reproduction [3,4] by affecting gene expression, protein synthesis and degradation, and membrane structure, as well as cytoskeleton dynamics [5,6]. In addition, heat can change the efficiency of intracellular enzymatic reactions, leading to internal metabolic imbalance, which in turn causes an excessive accumulation of toxic byproducts, such as reactive oxygen species (ROS) [7]. In order to shield the effects of heat stress on the internal metabolic processes, plants modulate the composition of corresponding transcripts, proteins, metabolites and lipids to establish a new metabolic homeostasis, as well as changing their growth and reproduction to cope with high-temperature environments [8,9].

High-throughput proteomics techniques have facilitated the large-scale identification of heat-responsive proteins (HRPs) in plants [9,10,11,12]. Proteomics data have revealed diverse expression patterns of HRPs in leaves of *Arabidopsis thaliana* [13], alfalfa (*Medicago sativa*) [10], *Oryza sativa* [14,15], *Oryza meridionalis* [16], wheat (*Triticum aestivum*) [17,18], maize (*Zea mays*) [19,20], soybean (*Glycine max*) [21,22], and *Apium graveolens* [23]. These HRPs are mainly involved in signal transduction, photosynthesis, ROS scavenging, transcription and post-transcriptional regulation, protein synthesis and degradation, as well as carbon and energy metabolism [13,14,15,16,17,18,19,20,21,22,23].

Spinach (*Spinacia oleracea* L.) is rich in vitamins, minerals and other nutrients, and is considered as one of the major green leafy vegetables in China. In general, spinach is a cold-tolerant but heat-sensitive species [24], and high temperatures cause a low germination rate of seeds and retarded growth, leading to a reduction of yield and nutrition. The investigation of heat-responsive molecular mechanisms in spinach will be instructive for breeding new varieties with heat tolerance capability. Previous studies have reported that heat stress (35 °C, 30 min) on whole spinach induced a significant decrease in the CO_2_ assimilation rate [25]. Heat stress also induced a release of the extrinsic oxygen evolving complex (OEC) subunits (PsbO, PsbP and PsbQ) from Photosystem II (PSII), which results in significant D1 aggregation and degradation [26,27]. Our previous proteomics study has identified 911 heat-responsive proteins in the heat-tolerant spinach variety Sp75 [11]. The data showed that calcium-mediated signaling, ROS homeostasis, endomembrane trafficking, and cross-membrane transport pathways were enhanced under heat stress. Moreover, diverse primary and secondary metabolic pathways (e.g., glycolysis, pentose phosphate pathway, isoprenoid biosynthesis, as well as metabolisms of amino acid, fatty acid, nucleotide, and vitamins) were employed for heat tolerance [11].

To compare and contrast with our previous study on the heat-tolerant spinach variety Sp75, a heat-sensitive variety Sp73 was subjected to heat-responsive physiological and proteomic analyses. The abundance patterns of 257 heat-responsive proteins imply that photosynthesis was inhibited, but ROS scavenging pathways, protein turnover, carbohydrate metabolism were enhanced in the heat-sensitive spinach variety Sp73. This study provides important insights into the molecular mechanisms of the heat stress response of spinach.

## 2. Results

### 2.1. Morphology and Relative Water Content (RWC)

**Figure 1:**
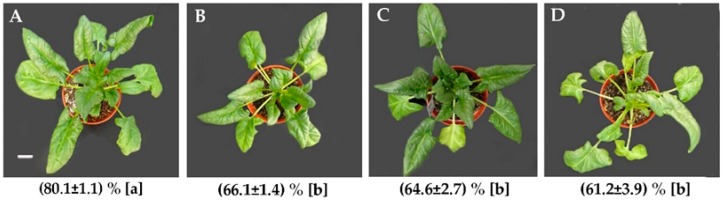
Morphology and relative water content (RWC) in spinach variety Sp73 under heat stress. Leaves were withered and the leaf RWC was decreased after 24 h of heat treatment (HHT) of 37 °C/32 °C (day/night). (**A**) 0 HHT; (**B**) 24 HHT; (**C**) 48 HHT; and (**D**) 72 HHT. Underneath each morphological image is the leaf RWC expressed as mean ± standard deviation (SD) (*n* = 3). Small letters (a, b) in the brackets indicate significant difference among different treatments (*p* < 0.05). Bar = 2.9 cm.

The morphology of leaves from spinach variety Sp73 were affected after 24 h of heat treatment (HHT), 48 HHT and 72 HHT, when compared with 0 HHT (Figure 1 A–D). The number of withered leaves increased, and the withering was more serious at 48 and 72 HHT (Figure 1C,D), although the length and width of leaves were not changed significantly. The RWC was decreased by 17.45%, 19.38% and 23.61% at 24 HHT, 48 HHT and 72 HHT, respectively. These results clearly show that spinach Sp73 is very sensitive to heat stress.

### 2.2. Photosynthesis and Chlorophyll Fluorescence Parameters

**Figure 2:**
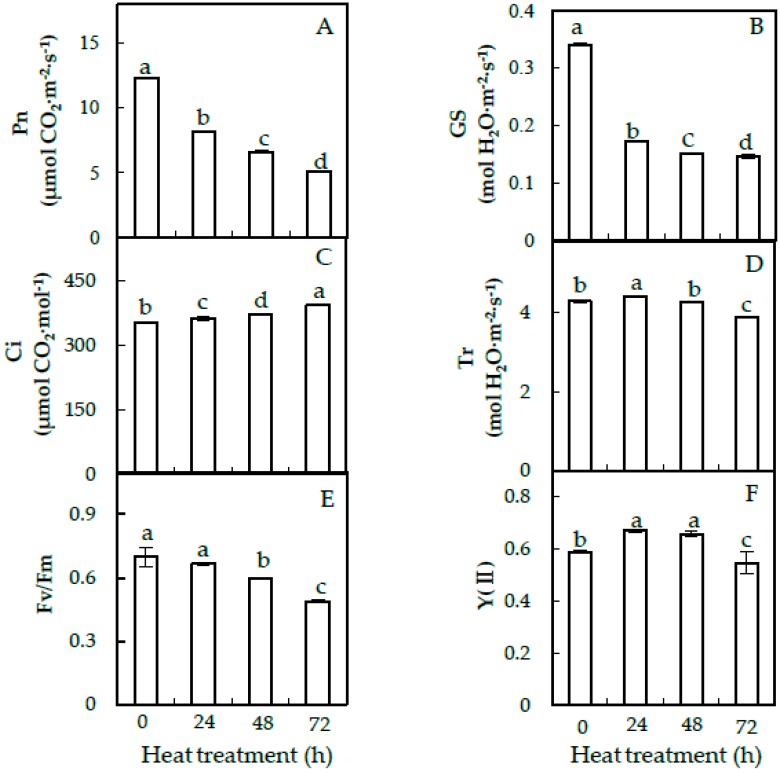
Photosynthetic characteristics and chlorophyll fluorescence parameters in leaves of spinach Sp73 under the heat-stress treatment. (**A**) Photosynthesis rate (Pn); (**B**) stomata conductance (Gs); (**C**) intercellular CO_2_ (Ci); (**D**) transpiration rate (Tr); (**E**) Photosystem II (PSII) maximum quantum yield (Fv/Fm); (**F**) effective PSII quantum yield (Y(II)). The values were determined after plants were treated with heat stress at 37 °C/32 °C (day/night) for 0 h, 24 h, 48 h and 72 h and are presented as means ± SD (*n* = 3). The different small letters (a–d) indicate significant difference (*p* < 0.05) in different treatments.

The photosynthesis rate (Pn), stomata conductance (Gs), intercellular CO_2_ (Ci) and transpiration rate (Tr) in heat-treated spinach Sp73 were measured (Figure 2). Compared with 0 HHT, the Pn (Figure 2A), Gs (Figure 2B) and Tr (Figure 2D) were apparently decreased by 2.4-fold, 2.3-fold and 1.1-fold at 72 HHT, respectively, throughout the heat-stress process. In addition, Ci was increased under the heat treatment process (Figure 2C). Furthermore, chlorophyll fluorescence parameters were also monitored to evaluate the photosynthetic performance. The PSII maximum quantum yield (Fv/Fm) was decreased 1.1-fold at 24 HHT, 1.2-fold at 48 HHT and 1.4-fold at 72HHT (Figure 2E). The effective PSII quantum yield (Y(II)) was slightly increased at 24 HHT and 48 HHT, but decreased at 72 HHT (Figure 2F).

### 2.3. Plasma Membrane Integrity and Osmolyte Accumulation in Leaves

**Figure 3:**
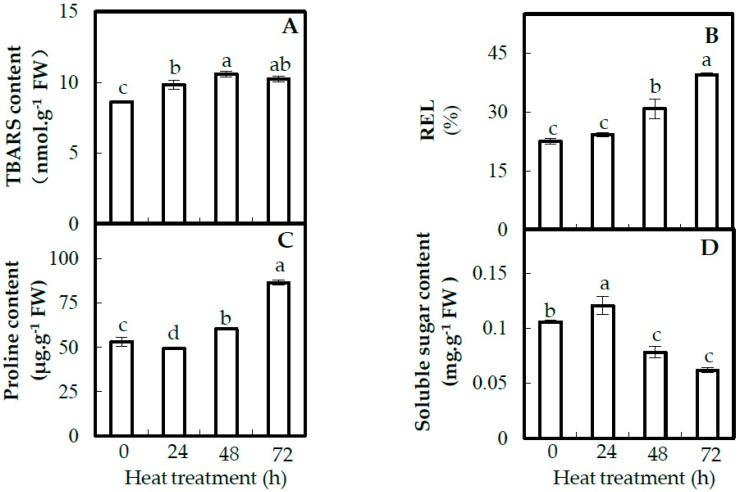
Membrane integrity and osmolyte accumulation in leaves of spinach Sp73 under heat stress. (**A**) Thiobarbituric acid reactive substance (TBARS) content in leaves; (**B**) relative electrolyte leakage (REL) of leaves; (**C**) proline in leaves; (**D**) soluble sugar content in leaves. The values were determined after plants were treated with heat stress at 37 °C /32 °C (day/night) for 0 h, 24 h, 48 h and 72 h and are presented as means ± SD (*n* = 3). The different small letters (a–d) indicate significant difference (*p* < 0.05) among different treatments.

To evaluate the effects of heat stress on membrane stability, the thiobarbituric acid reactive substance (TBARS) content and relative electrolyte leakage (REL) were detected in leaves under heat stress (Figure 3). TBARS contents increased from 8.6 ± 0.1 nmol∙g^−1^ fresh weight (FW) at 0 HHT to 10.6 ± 0.2 nmol∙g^−1^ FW at 48 HHT (Figure 3A). RELs were increased 1.4-fold at 48 HHT and 1.8-fold at 72 HHT compared with 0 HHT (Figure 3B). These results indicate that long-term heat stress leads to severe oxidative damage to leaf cells in spinach Sp73.

Proline and soluble sugars function by maintaining osmotic balance and protein stabilization. Compared with 0 HHT, the proline content in leaves was increased 1.6-fold at 72 HHT (Figure 3C). Soluble sugar contents were increased at 24 HHT, but decreased at 48 HHT and 72 HHT (Figure 3D).

### 2.4. Activities of Antioxidant Enzymes and Antioxidant Contents in Leaves

**Figure 4:**
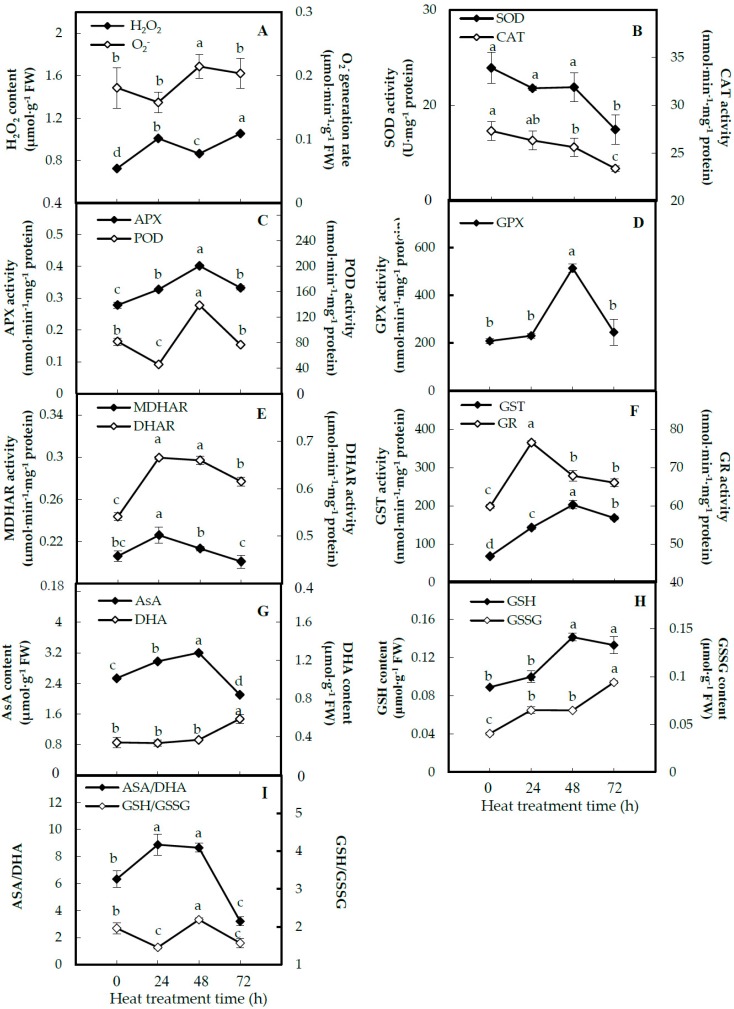
Activities of antioxidant enzymes and antioxidant contents in leaves of spinach Sp73 under heat-stress treatment. (**A**) Hydrogen peroxide (H_2_O_2_) content and superoxide anion radical (O_2_^•−^) generation rate; (**B**) activities of superoxide dismutase (SOD) and catalase (CAT); (**C**) activities of ascorbate peroxidase (APX) and peroxidase (POD); (**D**) glutathione peroxidase (GPX) activity; (**E**) activities of monodehydroascorbate reductase (MDHAR) and dehydroascorbate reductase (DHAR); (**F**) activities of glutathione reductase (GR) and glutathione S-transferase (GST); (**G**) contents of ascorbate (AsA) and dehydroascorbate (DHA); (**H**) contents of reduced glutathione (GSH) and oxidized glutathione (GSSG); (**I**) ratios of AsA/DHA and GSH/GSSG. The values were determined after plants were treated with heat stress at 37 °C/32 °C (day/night) for 0 h, 24 h, 48 h and 72 h and are presented as means ± SD (*n* =3). The different small letters (a–d) indicate significant difference (*p* < 0.05) among different treatments.

We analyzed the activities of antioxidant enzymes and antioxidant contents to evaluate the ROS changes and dynamics of the ROS scavenging system in leaves under the heat-stress treatment. Compared with 0 HHT, the hydrogen peroxide (H_2_O_2_) content and superoxide anion radicals (O_2_^•−^) generation rate were significantly increased in leaves at 48 HHT and 72 HHT (Figure 4A), implying that heat-increased ROS would cause oxidative damage to spinach leaves. The activities of superoxide dismutase (SOD) and catalase (CAT) were obviously decreased in the leaves throughout the heat-stress process (Figure 4B). However, the activities of ascorbate peroxidase (APX) and peroxidase (POD) were increased 1.9-fold and 1.7-fold at 48 HHT, respectively (Figure 4C). The activities of glutathione peroxidase (GPX) and glutathione S-transferase (GST) were increased 2.5-fold and 3.0-fold at 48 HHT, respectively (Figure 4D,F). Besides this, the activities of monodehydroascorbate reductase (MDHAR), dehydroascorbate reductase (DHAR) and glutathione reductase (GR) were all increased, except the MDHAR activity at 72 HHT, and all of them reached their highest level at 24 HHT and then decreased gradually at 48 HHT and 72 HHT (Figure 4E,F). For the antioxidants in the glutathione (GSH)-ascorbate (AsA) cycle, the AsA contents were decreased, while dehydroascorbate (DHA) contents were increased at 72 HHT (Figure 3G), and all the contents of reduced GSH and oxidized glutathione (GSSG) were increased in leaves under the heat-stress process (Figure 4H). The ratios of GSH/GSSG were decreased 1.3-fold and 1.2-fold at 24 HHT and 72 HHT, respectively, while they increased by 1.1-fold at 48 HHT (Figure 4I). However, the ratios of AsA/DHA were increased 1.4-fold at both 24 HHT and 48 HHT, while they decreased by 2.0-fold at 72 HHT.

### 2.5. Identification of Heat-Responsive Proteins by 2DE-Based and iTRAQ-Based Proteomics

**Figure 5:**
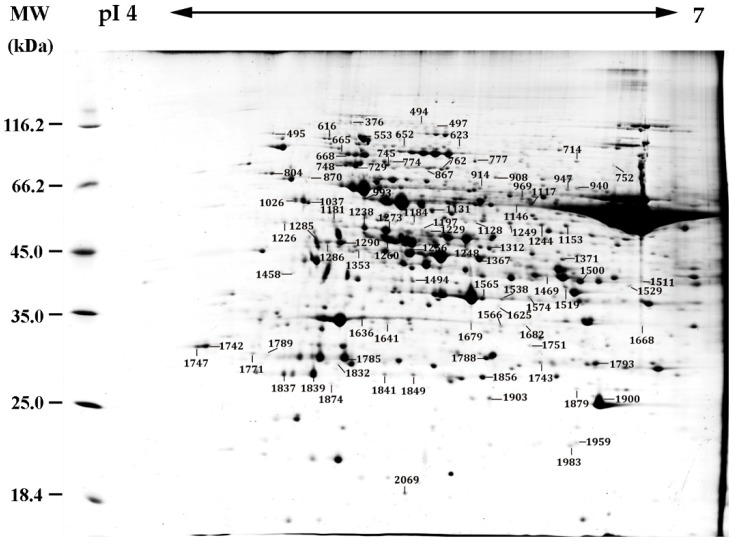
Representative two-dimensional gel electrophoresis (2DE) gel images of proteins in leaves of spinach Sp73. Proteins were separated on 24 cm linear gradient immobilized pH gradient (IPG) strips (pH 4–7) using isoelectric focusing (IEF) in the first dimension, followed by 12.5% sodium dodecyl sulfate polyacrylamide gel electrophoresis (SDS-PAGE) gels in the second dimension. The 2DE gel was stained with Coomassie Brilliant Blue. The molecular weight (MW) in kilodaltons (KDa) and isoelectric point (pI) of proteins are indicated on the left and top of the gel, respectively. A total of 84 heat-responsive proteins identified by matrix-assisted laser desorption/ionization tandem time of flight mass spectrometry (MALDI TOF-TOF MS) were marked with numbers on the gel. Detailed information can be found in Supplementary Figure S1 and Table S1.

**Figure 6:**
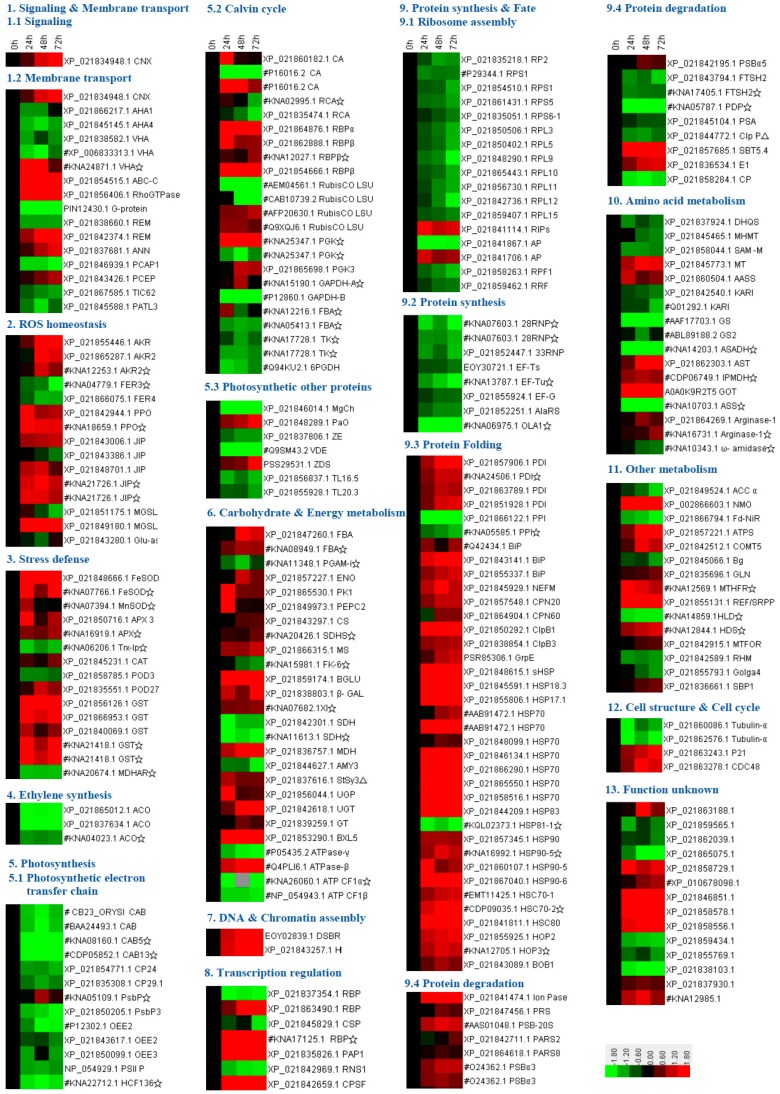
Abundance patterns of heat-responsive proteins in leaves of spinach Sp73 revealed from proteomic analysis. The four columns represent different heat treatments at 37 °C/32 °C (day/night) for 0 h, 24 h, 48 h, and 72 h. The rows represent individual proteins. The increased or decreased proteins are indicated in red or green, respectively. The color intensity increases with increasing abundance differences, as shown in the scale bar. The scale bar indicates the log (base2)-transformed relative protein abundance ratios ranging from −1.8 to 1.8. Database accession numbers and the abbreviations of protein names are listed on the right side (please refer to Supplementary Table S3 for the full protein names). The database accession numbers are from NCBInr. Those marked with the pound signs (#) indicate the proteins identified by the 2DE-based proteomics approach, and the rest were identified by the iTRAQ-based proteomics approach. The protein names marked with pentagrams (☆) were annotated according to the functional domain annotation from the NCBInr protein database.

Two complementary proteomics approaches, two-dimensional gel electrophoresis (2DE)-based and isobaric tags for relative and absolute quantification (iTRAQ)-based approaches, were applied to determine the heat-responsive protein abundances in leaves of spinach Sp73 under heat stress. From the results of 2DE-based proteomics, more than 1000 protein spots were detected on Coomassie Brilliant Blue (CBB)-stained gels (Figure 5, Supplementary Figure S1; Table S1). Among them, 93 reproducibly matched spots showed more than 1.5-fold changes in abundance in response to heat treatment (*p* < 0.05). Among them, 84 protein spots were identified by matrix-assisted laser desorption/ ionization tandem time of flight mass spectrometry (MALDI TOF-TOF MS) and Mascot searching with stringent criteria. The 84 protein spots all contained a single protein in each spot (Supplementary Table S1). Thus, the 84 proteins were taken as HRPs. Besides this, in the iTRAQ-based proteomics, 3526 proteins were identified and quantified in four independent biological replicates. Among them, 173 proteins showed differential abundances under heat stress (fold change > 1.2 and *p* < 0.05) (Supplementary Table S2). There were no overlaps between the results from 2DE-based and iTRAQ-based approaches. In total, 257 HRPs were identified in the leaves of spinach Sp73 (Figure 6, Supplementary Table S3). The HRPs were annotated against the National Center for Biotechnology Information non-redundant (NCBInr) protein database with Basic Local Alignment Search Tool (BLAST) analysis, and 51 proteins were reannotated according to the functional domain annotation from the NCBInr protein database (Supplementary Tables S4 and S5). Among these HRPs, 141 proteins were heat stress-increased and 112 heat-decreased under at least one heat stress condition compared with 0 HHT. The remaining four proteins exhibited different change patterns under heat stress.

### 2.6. Annotation and Functional Categorization of HRPs

**Figure 7:**
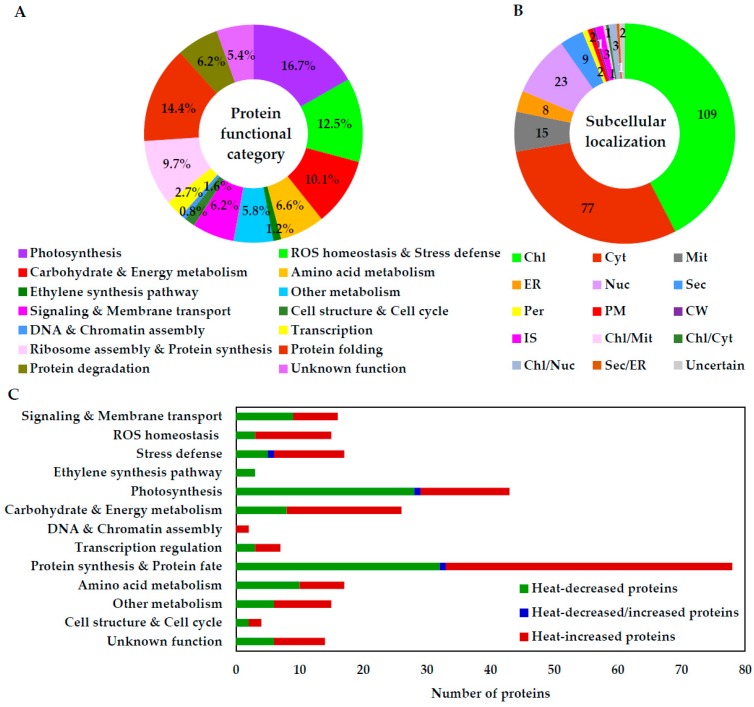
Functional categorization and subcellular localization of heat-responsive proteins. (**A**) Protein functional categories; (**B**) subcellular localization groups. The numbers of proteins with different locations are shown; (**C**) abundance patterns of heat-responsive proteins in each functional category. Chl, chloroplast; CW, cell wall; Cyt, cytoplasm; ER, endoplasmic reticulum; IS, intercellular space; Mit, mitochondrion; Nuc, nucleus; Per, peroxisome; PM, plasma membrane; Sec, secreted.

Based on BLAST alignments, subcellular localization prediction, and literature information, the 257 proteins were classified into 13 functional categories, including signaling and membrane transport, ROS homeostasis, stress defense, ethylene synthesis, photosynthesis, carbohydrate and energy metabolism, DNA and chromatin assembly, transcription regulation, protein synthesis and fate, amino acid metabolism, other metabolisms, cell structure and cell cycle, and unknown function (Supplementary Table S3 and Figure 7A). Interestingly, protein synthesis and fate-related proteins accounted for the largest group (30.4%), followed by photosynthesis-related proteins (16.7%). A total of 54.1% of HRPs were heat-increased compared with 0 HHT (Figure 7C). For example, 12 out of 15 (80%) ROS scavenging proteins (e.g., SOD, APX, and GST) and 11 out of 17 (64.7%) stress defense-related proteins (e.g., aldo/keto reductase (AKR) and jasmonate-induced protein) were heat stress-increased. Besides this, carbohydrate and energy metabolism-related proteins (69.2%) and protein turnover-related proteins (57.7%) were increased by heat stress. Interestingly, 83.0% of protein folding/degradation-related proteins were increased in the heat-sensitive spinach Sp73. In contrast, most proteins involved in photosynthesis (65.1%), amino acid metabolism (58.8%), ribosome assembly (92.0%) and protein synthesis (100%) were decreased by heat stress. These results indicate that carbon assimilation, basic metabolism and protein synthesis were inhibited by heat stress in the heat-sensitive spinach Sp73.

### 2.7. Subcellular Localization and Protein–Protein Interaction (PPI) Network of HRPs

The subcellular localization of HRPs was predicted using five different tools (*i.e*., YLoc, LocTree3, Plant-mPLoc, ngLOC, and TargetP) (Figure 7B, and Supplementary Table S6). Among the 257 proteins, 109 proteins (42%) were predicted to be localized in chloroplasts, 77 (30%) in cytoplasm, 23 (9%) in nucleus, 15 (6%) in mitochondria, nine in the secreted pathway, and eight in the endoplasmic reticulum. The remaining proteins are localized in the plasma membrane (2), intercellular space (3), cell wall (1), peroxisomes (2) and uncertain locations (2). Clearly, most heat-affected proteins were localized in chloroplasts, indicating the heat-stress sensitivity of the chloroplasts in the spinach Sp73.

**Figure 8:**
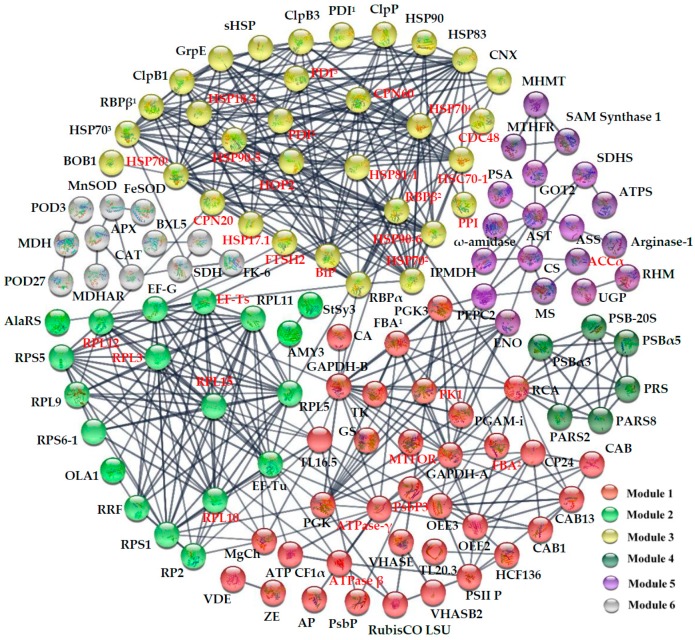
The protein–protein interaction (PPI) network of heat-responsive proteins in spinach Sp73 revealed by functional protein association networks (STRING) analysis. A total of 123 unique homologous proteins from *Arabidopsis thaliana* are shown in the PPI network. Six modules are indicated in different colors. Module 1: photosynthesis; Module 2: protein synthesis; Module 3: protein folding; Module 4: protein degradation; Module 5: sugar metabolism, amino acid metabolism, and other metabolisms; Module 6: ROS scavenging and stress defense. The PPI network is shown in the confidence view generated by STRING analysis. Stronger associations are represented by thicker lines. Please refer to Supplementary Table S7 for abbreviations.

A total of 207 unique homologous proteins of HRPs were found in Arabidopsis (Supplementary Table S7), 123 of which were depicted in the PPI network. Six modules formed tightly connected clusters, and strong associations were represented by thick lines in the network (Figure 8). Module 1 contained 35 proteins mainly involved in photosynthesis. Module 2, Module 3 and Module 4 included those proteins mainly involved in protein synthesis, protein folding and protein degradation, respectively. Module 5 contained 19 proteins which are mainly involved in sugar metabolism, amino acid metabolism and other metabolisms. Module 6 contained 12 proteins important in ROS scavenging and stress defense.

## 3. Discussion

### 3.1. Heat-Inhibited Photosynthesis in Heat-Sensitive Spinach Variety Sp73

Heat stress has a negative impact on the photosynthetic capacity of plants [8], such as grape (*Vitis vinifera*) [28], wheat [17] and soybean [22]. Photosynthetic reactions which occur in thylakoid membranes (both stacked grana and lamellae) and carbon metabolism in the stroma of chloroplasts are primary sites of heat damage [29]. In general, spinach is a heat-sensitive vegetable species. Through genetic screening, we identified a heat-tolerant variety, Sp75 [11]. Our results in this study showed that photosynthesis was decreased in both the heat-tolerant variety Sp75 and heat-sensitive variety Sp73 under heat stress (Figure 2). Compared with the heat-tolerant Sp75, the heat-sensitive Sp73 exhibited obvious decreases in photosynthetic parameters, such as Pn and Gs, throughout the heat-stress process (Figure 2; Supplementary Figure S2).

The PSII is highly thermolabile and its activity is greatly diminished at high temperature [30], and this decrease may be due to PSII localization in the thylakoid membrane [31]. Our proteomics results showed that the abundances of most PSII proteins (e.g., chlorophyll a-b binding protein, oxygen-evolving enhancer protein, OEC subunit PsbP, and high chlorophyll fluorescence 136) were all decreased in the Sp73 under heat stress process. This indicates that OEC might be dissociated and the potential active center of PSII was damaged in the heat-sensitive spinach Sp73 [32]. This is similar to that found in heat-sensitive *A. stolonifera* [33], leading to the inhibition of light harvesting and electron transport under heat stress [34].

Moreover, we identified that 11 of the 19 HRPs in the Calvin cycle were decreased in the Sp73, resulting in inhibited carbon assimilation (Figure 6) [29]. This is consistent with previous studies in grape [35] and soybean [21]. Among the Calvin cycle enzymes, ribulose bisphosphate carboxylase/oxygenase (RuBisCO) and RuBisCO activase (RCA) were reported to be very sensitive to high temperature and showed diverse abundance patterns. For example, RCA in the wild downy grape leaves was decreased [35]. However, RCAs in *A. thaliana* [13] and rice [14] leaves were increased in response to heat stress. RCA is a molecular chaperone that plays an important role in maintaining the catalytic activity of RuBisCO [36] and is one of the limiting factors for photosynthesis under heat stress [37,38]. In addition, enzymes involved in chlorophyll synthesis (e.g., magnesium-chelatase subunit ChlI) and carotenoid biosynthesis (e.g., zeta-carotene desaturase) were decreased under heat stress. On the other hand, pheophorbide oxygenase involved in chlorophyll degradation was increased in the Sp73. This is quite different from the nine heat-increased chlorophyll biosynthetic enzymes in the heat-tolerant Sp75 (Supplementary Figure S3 and Table S8). This implies that the heat-sensitive Sp73 has a lower capability of chlorophyll maintenance than Sp75 for photosynthesis under heat stress [11].

In addition, the xanthophyll cycle is usually regarded as the most important photoprotection mechanism in higher plants. In this cycle, violaxanthin de-epoxidase and zeaxanthin epoxidase are critical enzymes. In Sp73, violaxanthin de-epoxidase and zeaxanthin epoxidase were decreased, indicating the photoprotection of the reaction center of PS II was reduced under heat stress [39].

### 3.2. Heat-Altered ROS Scavenging Pathways in Spinach Sp73

In spinach Sp73 leaves, the accumulation of H_2_O_2_ was obviously induced by heat stress, leading to membrane lipid peroxidation and damage (Figure 3) [40]. The accumulated ROS may be due to the low electron transport efficiency under heat stress [34]. Here, we found that various anti-oxidative enzymes and antioxidants were altered in intracellular H_2_O_2_ scavenging to cope with heat stress (Figure 4). Among them, the SOD activity was decreased by heat stress, in spite of the increased abundance of chloroplast-/mitochondrion-localized SODs under heat stress. This suggests that the first line of heat response to dismutate the intracellular O_2_^•−^ to H_2_O_2_ was inhibited in the Sp73 [41], while the activities and abundances of SOD in the heat-tolerant Sp75 (Supplementary Figure S3) [11] and other heat-tolerant species (e.g., maize [20], poplar (*Populus yunnanensis*) [42] and wheat [43,44] were increased by heat stress. Interestingly, other antioxidant enzymes involved in converting intracellular H_2_O_2_ to H_2_O showed distinct activity patterns under heat stress. Among them, CAT activities were decreased, while APX and GPX activities were increased under the heat-stress process. The activity changes of CAT and APX in the Sp73 were opposite to those in the Sp75 (Supplementary Figure S2), indicating the activation of different antioxidant enzymes to scavenge ROS between the heat-sensitive Sp73 and heat-tolerant Sp75 [11]. Besides this, the accumulation of DHA, GSH and GSSG in the AsA-GSH pathway under the heat-stress process was observed in Sp73, similar to what happened in the heat-tolerant Sp75 (Supplementary Figure S3), maize [20], wheat [43] and poplar [42] under heat stress. It appears that the AsA-GSH pathway is conserved in plants to remove the heat-induced H_2_O_2_ [11]. Another similar strategy between the Sp73 and Sp75 is the increased abundances of GST and AKR (Supplementary Figure S3 and Table S8), which can contribute to detoxifying lipid peroxidation-derived reactive aldehydes and lead to enhanced salt-stress tolerance [45].

In addition, the accumulation of non-enzymatic antioxidants (e.g., proline and soluble sugars) can help to buffer redox changes and stabilize subcellular structures [46]. This is an effective strategy for dealing with heat stress-induced oxidative stress [47]. In the heat-tolerant Sp75, the contents of proline and soluble sugars were increased in response to heat stress (Supplementary Figure S2). Similar cases were also observed in tomato (*Lycopersicon esculentum*) [48], tobacco (*Nicotiana tabacum*) [49] and poplar [42], indicating that the heat stress-induced accumulation of soluble sugars and proline plays an important role in heat-stress tolerance. However, the soluble sugar content was decreased in the Sp73. This result may explain the heat sensitive phenotype of the Sp73.

### 3.3. Heat-Stress Signaling and Transport Pathways in Spinach Sp73

Plasma membrane fluidity and calcium ion channels are disturbed under high temperatures, leading to the entry of calcium ions into cells. Calcium ions can also be released from intracellular calcium stores, thereby activating calcium-dependent signal transduction pathways in response to high temperatures [4]. Our previous proteomics investigation revealed that several signaling proteins (e.g., calcium-dependent protein kinase 3, multiprotein bridging factor 1, dehydration-responsive element-binding, and 14–3-3 protein) were accumulated and the abundance of protein phosphatase 2C (a negative regulator in the mitogen-activated protein kinase (MAPK) pathway) was decreased by heat stress (Supplementary Figure S3 and Table S8), indicating the induction of a calcium-mediated MAPK cascade in the heat-tolerant Sp75 [11]. Besides this, several transporters involved in the transport of water, ions and metabolites were increased in the heat-tolerant Sp75 under high-temperature stress (Supplementary Figure S3 and Table S8) [11]. However, in the heat-sensitive Sp73, only a few calcium-related proteins and transporters were detected to be changed in levels. Among them, only annexin (ANN) and adenosine-triphosphate (ATP)-binding cassette (ABC) transporters were increased by heat stress. ANN is a calcium-dependent membrane-bound protein with ion channel activity. Its important role in the adaptation of plant cells to osmotic stress has been demonstrated [15]. AtANN1 is important in regulating the heat stress-induced [Ca^2+^]_cyt_ in Arabidopsis seedlings [18]. ATP-binding cassette transporters carry out the transmembrane transport of various biomolecules using the energy generated from hydrolyzing ATP. They play an important role in cellular detoxification, plant growth and development, and pathogen defense [28]. In addition, four H^+^-ATPases were heat-decreased, indicating the decrease of trans-membrane proton movement, and the perturbed membrane potential and secondary solute transport in the heat-sensitive Sp73 [50,51].

### 3.4. Heat Stress-Perturbed Diverse Primary and Secondary Metabolisms

Carbohydrate metabolism plays an important role in plant growth, development and stress response [52]. Our proteomic results indicate that the carbohydrate metabolism tends to be induced in the heat-sensitive Sp73 under heat stress. For example, 18 out of 26 enzymes involved in glycolysis, the tricarboxylic acid (TCA) cycle, and other sugar metabolism were heat-increased. This result is similar to the heat-tolerant Sp75 (Supplementary Figure S3 and Table S8) [11]. It was also reported that fructose-bisphosphate aldolases (FBAs) in alfalfa seeds [10], wild downy grape leaves [35], and enolases in wheat spikelets and seeds were increased under heat stress [53,54]. In addition, we found here that a starch synthase was increased but an alpha amylase was decreased, which would favor starch accumulation and tolerance to heat stress [55].

Amino acid metabolism showed differential change patterns between the heat-sensitive Sp73 and the heat-tolerant Sp75 under heat stress (Supplementary Figure S3 and Table S8). In Sp73, 10 out of 17 enzymes involved in amino acid metabolism were heat-decreased. Among them, glutamine synthetase (GS) catalyzing the assimilation of ammonium to glutamine was significantly decreased, which would reduce the nitrogen metabolism in spinach under heat stress. This is consistent with the findings in *Agrostis* grass [56] and wheat [18]. In contrast, most of the amino acid metabolic enzymes (76%) in Sp75 were obviously increased under heat stress (Supplementary Figure S3 and Table S8). These results indicate that the enhancement of amino acid metabolism is critical for heat-stress tolerance [11].

### 3.5. Heat Stress-Induced Transcriptional Regulation and Protein Processing in Sp73

In the heat-stressed Sp73, the repair of chromosome/DNA and transcriptional regulation might be enhanced, because two crucial proteins (high-mobility group-Y-related protein A-like and DNA double-strand break repair rad50 ATPase) were significantly increased to facilitate nucleosome formation, transcriptional regulation [57], telomere maintenance, and DNA damage checkpoint control [58]. Besides this, we found that nucleus-localized RNA-binding protein family members (RBPs) and poly(A) polymerase were increased, but chloroplast-localized RBP was decreased in the Sp73 under stress. These results indicate that the RNA stability, maturation, and transport in nucleus were enhanced, but were decreased in chloroplasts when Sp73 experienced heat stress. In addition, our proteomics results indicated that protein synthesis was inhibited in the Sp73. A number of small ribosomal subunits (e.g., RP, S1, S5 and S6) and large ribosomal subunits (e.g., L3, L5, L9, L10, L11, L12 and L15), as well as translation-related factors (e.g., elongation factors Tu, elongation factors Ts and elongation factors G) were decreased. In contrast, ribosome-inactivating proteins and rRNA N-glycosidase proteins, which inactivate ribosomes, were increased in the Sp73 under heat stress.

Importantly, we found that 34 out of 37 (92%) proteins involved in protein folding and processing were significantly increased in Sp73 under heat stress, including heat shock protein 70 (HSP70), heat shock cognate 70 kDa protein 1 (HSC70–1), HSP70–2, HSP83, HSP90–5, HSP70-HSP90 organizing protein, chaperonin (Cpn), protein GrpE, and small HSPs. The heat-enhanced protein folding and processing in Sp73 were consistent with Sp75 (Supplementary Figure S3 and Table S8). These increased proteins may help to prevent proteins from improper folding, denaturation, and aggregation under heat stress [59]. It has been reported that the HSP70 family can interact with HSP90 to promote protein folding and maintain a stable structure under heat stress [60], and Cpn60 can protect RuBisCO activase from thermal denaturation and function in acclimating photosynthesis under heat stress [61]. A significant increase in the abundances of Cpn60, HSP70, HSP90, and small HSPs were also found in rice [14,15], soybean [21], purslane (*Portulaca oleracea*) [62], and *C. spinarum* [63]. For example, HSP70s, chaperonins and small HSPs were increased in rice seedlings at 45 °C for 48 h [14] and in rice leaves under 42 °C for 12 h and 24 h [15]. Besides this, HSP70, heat shock cognate (HSC) 70, and several low molecular weight HSPs (e.g., HSP22, HSP18.5, and HSP 17.5) were newly induced and/or highly increased in soybean leaves, stems, and roots under 40 °C [21]. Moreover, HSP70, HSP90, and the molecular chaperones DnaK and DnaJ were all increased in purslane under 35 °C [62]. Importantly, the HSPs and chaperonins accounted for the largest category (43.3%) of heat-responsive proteins in *C. spinarum* under 35 °C treatment. Among them, small HSPs were remarkably induced [63]. These results suggest the increased abundances of molecular chaperones are necessary for thermos-tolerance [64]. In addition, four protein disulfide-isomerases contributing to the formation of natural disulfide bonds were significantly increased under heat stress, allowing proteins to enter the normal folding pathway under heat stress.

In addition, there is an active protein degradation pathway to prevent the accumulation of non-functional or potentially toxic proteins in heat-stressed spinach leaves. In Sp73, ten proteins associated with protein degradation were increased under heat stress; e.g., ubiquitin-activating enzyme E1, ion protease, cysteine protease, 26S proteasome proteins, and 20S proteasome proteins. Interestingly, the abundance of ion protease was increased 23-fold at 48 h under heat stress, which can help to maintain the cellular protein turnover by mediating the abnormal or transient regulation of protein degradation. This is consistent with that shown in Sp75 (Supplementary Figure S3 and Table S8) [11].

## 4. Materials and Methods

### 4.1. Plant Cultivation and Treatment

Seeds of spinach (*Spinacia oleracea* L.) variety Sp73 were collected from the Germplasm Resources Center of Shanghai Normal University. Seeds were sown on the sterilized culture matrix and grown in a growth chamber with a 22 °C / 18 °C and 10 h / 14 h day/night cycle, and 60% relative humidity for 50 days. Plants were watered daily to avoid the occurrence of water deficit. On the 50th day, plants of the treatment groups were moved to another growth chamber with the same growth condition as the control, except for temperature (37 °C / 32°C day/night), and watered daily on a regular schedule as well. The spinach Sp73 were treated for 0 h, 24 h, 48 h and 72 h. After plant morphological changes were recorded, fully expanded true leaves were collected for both control and heat-treated plants, immediately frozen in liquid nitrogen and stored at −80 °C for future physiological and proteomics analyses. For each treatment, at least three biological replicates were performed [11].

### 4.2. RWC Measurement

To determine RWC, 0.2 g of fresh leaves were detached, weighed immediately and recorded as fresh weight (A). Subsequently, the leaves were then immersed in distilled water for 24 h, the turgid weight (B) was quickly measured, and they were dried at 80 °C for 2 h and then 60 °C to constant weight, and the dry weight (C) was recorded. The RWC as calculated as follows: RWC = [(A −C) / (B − C)] × 100% [11].

### 4.3. Photosynthesis and Chlorophyll Fuorescence Parameter Measurement

Photosynthetic parameters (Gs, Ci, Pn, and Tr) were measured on fully expanded leaves of each plant using a portable photosynthesis system LICOR 6400 (LI-COR Inc., Lincoln, NE, USA) [65]. Fv/Fm and Y (II) were determined using OS5p+ (Model OS5p+, OPTI-Sciences, Hudson, NH, USA). After the dark adaptation of spinach leaves for 0.5 h, Fv/Fm was measured 4 times. Y(II) was measured 4 times using a red-light source.

### 4.4. Determination of TBARS Content, REL, Total Soluble Sugar, and Proline Contents

Lipid peroxidation was measured as the amount of TBARS determined by the thiobarbituric acid reaction according to Lee et al. [15,66]. TBARS was extracted from fresh leaves in 10% trichloroacetic acid and 0.6% thiobarbituric acid solution under 100 °C for 5 min. The absorbance of the supernatant at 450 nm, 532 nm, and 600 nm was detected as OD450, OD532 and OD600, respectively. The TBARS concentration was calculated according to the following equations: C (nmol L^−1^) = 6.45 × (OD532−OD600) − 0.56 × OD450; TBARS concentration (nmol∙g^−1^ **FW**) =C × V/FW (V, volume of total extraction solution; FW, fresh weight of leaves).

The REL was determined as described by Wang et al. [66]. The electrical conductivity of deionized water (E0) was detected at room temperature, which was measured using a conductivity instrument (DDS-11A). The fresh leaves were cut and immersed in 20 mL deionized water, and then were incubated at 100 °C for 10 min. The electrical conductivity of samples before and after boiling were recorded as E1 and E2. The REL was calculated according to the equation REL (%) = (E1 − E0) / (E2 − E0) × 100%.

The contents of soluble sugars and proline were measured using ninhydrin reaction and sulfuric acid–anthrone reagents according to a previous report [66]. For soluble sugar assay, the fresh leaves were ground in deionized water and incubated at 100 °C for 30 min. The supernatant was collected and mixed with 2% (*w*/*v*) ethyl acetate solution of anthrone and concentrated sulfuric acid, and then was incubated at 100 °C for 1 min. After cooling down to room temperature, the absorbance of the solution was measured at 630 nm using a spectrophotometer. Soluble sugar concentration was calculated with the concentration (C) determined from glucose standard curves, volume of total extracion solution (V), and fresh weight of leaves (FW) according to the following equation: soluble sugar concentration (mg∙g^−1^ FW) = C × V/FW. For proline assay, the fresh leaves were ground in 3% sulfosalicylic acid and incubated at 100 °C for an hour. The supernatant was collected after being centrifuged at 15,000 rpm for 5 min. Then, 1 mL supernatant, 2 mL glacial acetic acid, 2 mL ninhydrin were mixed and boiled for 30 min. After cooling down, 4 mL methylbenzene was added into the mixture and stood for two hours. The upper layer was collected for absorbance reading at 520 nm using a spectrophotometer. The proline concentrations in samples were calculated with concentration (C), volume of total extraction solution (V), and fresh weight of leaves (FW): proline concentration (μg∙g^−1^ FW) = C × V/FW.

### 4.5. Determination of ROS and Antioxidant Substance Contents, and Antioxidant Enzyme Activity Assay

The H_2_O_2_ content in leaves was measured according to the method of Ibrahim et al. [67]. The leaves were ground with 0.1% trichloroacetic acid and then centrifuged at 15,000× *g* for 15 min at 4 °C. The supernatant was collected and determined spectrophotometrically at 390 nm after reacting with potassium iodide. To determine the O_2_^−^ generation rate and antioxidant enzyme activities, 0.2 g frozen leaves were ground in an extraction buffer containing 50 mM phosphate buffer solution (pH 7.8), 2% polyvinylpyrrolidone-40, and 2 mM reduced ascorbate (AsA) (for ascorbate peroxidase (APX) activity assay) at 4 °C. After centrifugation at 20,000× g for 15 min at 4 °C, the supernatant was collected for analysis. The O_2_^•−^ generation rate was measured using a hydroxylamine oxidization method [68].

The activities of six antioxidant enzymes (SOD, CAT, POD, APX, GPX and GST) were determined according to the method of Yin et al. [68]. SOD activity was assayed on the basis of its ability to inhibit the photochemical reduction of nitro blue tetrazolium (NBT) at 560 nm. One unit of SOD activity was defined as the amount of enzyme that inhibited 50% of NBT photoreduction [68]. CAT activity was assayed by measuring H_2_O_2_ consumption at 240 nm [68]. POD activity was determined by a guaiacol method at 470 nm [65]. APX activity was measured by monitoring the absorbance decrease at 290 nm as the ascorbate was oxidized [20]. GPX activity was measured by recording the absorbance changes at 340 nm because of the oxidation of NADPH [69]. GST activity was measured by the product of CDNB conjugated with GSH absorbed at 340 nm [65]. The activities of MDHAR, DHAR and GR were measured by recording the absorbance changes at 340 nm due to the oxidation of NADH, at 265 nm due to the production of oxidized glutathione (GSSG) (ε = 14 mM^−1^·cm^−1^), and at 340 nm due to the oxidation of NADPH (ε = 6.22 mM^−1^·cm^−1^), respectively. Their activities were subsequently expressed as the amount of NADH oxidized, GSSG produced, and NADPH oxidized per minute per milligram protein, respectively [68]. For the contents of AsA and DHA, total AsA and reduced AsA were determined by recording the absorbance changes at 525 nm [68]. DHA content was estimated from the difference between assays with and without dithiothreitol (DTT) [68].

### 4.6. Quantitative Proteomics Analyses

The proteins were extracted using a phenol extraction method according to Yu et al. [65]. The protein pellet was dissolved in a lysis solution (7 M urea, 2 M thiourea, 4% 3-[(3-Cholamidopropyl) dimethyl-ammonio] propanesulfonic acid (CHAPS), 0.04 M DTT, and 4% protease inhibitor cocktail). Protein concentration was determined using a 2D Quant Kit according to the manufacturer’s instructions (GE Healthcare, Uppsala, Sweden).

For 2DE-based proteomics analysis, about 1.6 mg protein extract was loaded into per gel, separated on linear gradient IPG strips (24 cm, pH 4–7) through isoelectric focusing (IEF) in the first dimension, then transferred into 12.5% SDS-PAGE for two-dimensional electrophoresis, and stained by Coomassie Brilliant Blue staining. Gel image acquisition and analysis were conducted as described in detail by Wang et al. [66]. The volume of each spot was normalized to the total volume of all the detected spots. Protein spots considered to be differential abundant proteins needed to show consistent abundance changes from three biological replicates with greater than 1.5-fold changes and a *p* value smaller than 0.05 [65]. In-gel digestion was performed on the protein spots with abundance differences. MS was calibrated using a quality standard kit (AB Sciex Inc., Frammingham, MA, USA) and a bovine serum albumin standard (Sigma-Aldrich, St. Louis, MO, USA). MS/MS spectra were obtained using an ABI 5800 MALDI TOF/TOF MS (AB Sciex, Foster City, CA, USA). The mass spectrum error of MS and MS/MS was less than 30 ppm, and the resolution was 10,000. To determine the confidence of the protein search results, the following criteria were applied: (1) top-ranked search results (top five results); (2) the probability score obtained by molecular weight searching (MOWSE) should be greater than 50 (*p* < 0.01); (3) at least two peptide matches, all Y-ion series and partially complementary B-ion series should correspond to the high-intensity peaks.

For iTRAQ-based proteomics analysis, the protein samples of spinach leaves treated for 0 h, 24 h, 48 h and 72 h were digested using trypsin (1:50, *w*/*w*, trypsin: sample). The digested samples were labeled with iTRAQ reagents 113 & 117 (0 h), 114 & 118 (24 h), 115 & 119 (48 h), and 116 & 121 (72 h). Then, different samples were mixed together. The peptide mixture was fractionated on XBridge C18 column (150 mm × 4.6 mm, 5 μm, Waters, Milford, MA, USA) using a Shimadzu LC-20A high-performance liquid chromatography (HPLC) system (Shimadzu, Kyoto, Japan). Each of the fractionated components was desalted with 3 M Empore C18 solid-phase extraction disks (3 M Bioanalytical Technologies, St.Paul, MN, USA). Peptide samples were identified by an online nanoacquity ultraperformance LC (Waters, Milford, MA, USA) coupled with an Orbitrap Fusion Tribrid mass spectrometer (Thermo Fisher Scientific, San Jose, CA, USA) using a method by Zhao et al. [11]. The MS2 spectra obtained from the MS analysis were searched against a protein database [70] via ProteinPilot Software 4.5 (AB Sciex, Frammingham, MA, USA). The credible protein identification and quantitative results needed to meet the following criteria: unused protein score > 1.3 and number of unique peptides ≥ 2. In at least three biological replicates, the proteins with fold changes >1.2 and *p* < 0.05 in the treatment group compared to the control group were defined as HRPs.

### 4.7. Protein Function Classification and Cluster Analysis

Protein functional domains were analyzed by BLAST alignment against the NCBInr protein database [71], and the proteins were classified into different functional groups by combining with the knowledge from the Kyoto Encyclopedia of Genes and Genomes (KEGG) pathway database [72], UniProt database [73], Gene Ontology database [74], and literature information.

### 4.8. Protein Subcellular Localization Prediction

Protein subcellular localization was predicted with five different online tools: YLoc [75], LocTree3 [76], ngLOC [77], Plant-mPLoc [78], TargetP [79]. Only the consistent predictions from at least two tools were accepted as confident results listed in the column of “confirmed localization”.

### 4.9. Protein-Protein Interaction Prediction

The homologs of spinach heat-stress response proteins in Arabidopsis were obtained by sequence BLAST alignment in the Arabidopsis Information Resource (TAIR) database. The homologs were used in the Web tool of STRING 10.5 [80] to create the PPI network.

### 4.10. Statistical Analysis

Each physiological/proteomics experiment result was obtained from at least three biological replicates. The experimental results were listed as mean ± standard deviation. The differences between different treatment samples in respect of physiological indexes were compared using one-way analysis of variance (one-way ANOVA) in SPSS 17.0. The differences between individual treatment group and the control group in respect of the protein abundance were compared using the Student *t*-test, and *p* < 0.05 was considered to be statistically significant.

## 5. Conclusions

In this study, the physiological and proteomic changes in the heat-sensitive spinach Sp73 were reported and compared with those in the heat-tolerant spinach Sp75. Interestingly, the two spinach varieties possess some common thermal response processes, but also show specificity in response to heat stress. Our results revealed that photosynthesis was heat stress-inhibited, but ROS scavenging pathways and stress defense, carbohydrate and energy metabolism, and protein folding and degradation were heat stress-enhanced in both Sp73 and Sp75, implying the conservation of these processes in the response of spinach to high-temperature stress. Notably, calcium signaling, endomembrane trafficking, as well as the regulation of the cell cycle and differentiation were specifically enhanced in heat-treated spinach Sp75. Moreover, signal transduction, protein synthesis, and amino acid metabolism were heat stress-suppressed in spinach Sp73 but enhanced in Sp75. All these data provide important insights into the molecular mechanisms underlying the heat-stress response/tolerance of the two contrasting spinach varieties Sp75 and Sp73.

## Supplementary Materials

Supplementary materials can be found at https://www.mdpi.com/1422-0067/20/16/3872/s1.

## Abbreviations

2DE	Two-dimensional gel electrophoresis
AKR	Aldo/keto reductase
ANN	Annexin
APX	Ascorbate peroxidase
AsA	Ascorbate
ATP	Adenosine-triphosphate
BLAST	Basic Local Alignment Search Tool
CAT	Catalase
Ci	Intercellular CO_2_
DHA	Dehydroascorbate
DHAR	Dehydroascorbate reductase
Fv/Fm	PSII maximum quantum yield
FW	Fresh weight
GPX	Glutathione peroxidase
GR	Glutathione reductase
Gs	Stomata conductance
GSH	Reduced glutathione
GSSG	Oxidized glutathione
GST	Glutathione S-transferase
H_2_O_2_	Hydrogen peroxide
HHT	Hour of heat treatment
HRP	Heat stress-responsive protein
IEF	Isoelectric focusing
IPG	Immobilized pH gradient
iTRAQ	Isobaric tags for relative and absolute quantification
MALDI	Matrix-assisted laser desorption/ ionization
MAPK	Mitogen-activated protein kinase
MDHAR	Monodehydroascorbate reductase
MS	Mass spectrometry
NCBInr	National Center for Biotechnology Information non-redundant
O_2_^•−^	Superoxide anion radicals
OEC	Oxygen evolving complex
pI	Isoelectric point
Pn	Photosynthesis rate
POD	Peroxidase
PPI	Protein-protein interactions
PSII	Photosystem II
RBP	RNA-binding protein family member
RCA	Ribulose bisphosphate carboxylase/oxygenase activase
REL	Relative electrolyte leakage
ROS	Reactive oxygen species
RubisCO	Ribulose-1,5-bisphosphate carboxylase/oxygenase
RWC	Relative water content
SDS-PAGE	Sodium dodecyl sulfate polyacrylamide gel electrophoresis
SOD	Superoxide dismutase
Sp73	Heat-sensitive spinach
Sp75	Heat-tolerant spinach
STRING	Search tool for recurring instances of neighbouring genes
TBARS	Thiobarbituric acid reactive substance
TOF	Time of flight
Tr	Transpiration rate
Y(II)	Effective PSII quantum yield
